# Plastid ribosome protein L5 is essential for post-globular embryo development in *Arabidopsis thaliana*

**DOI:** 10.1007/s00497-022-00440-9

**Published:** 2022-03-05

**Authors:** Gilles Dupouy, Emma McDermott, Ronan Cashell, Anna Scian, Marcus McHale, Peter Ryder, Joelle de Groot, Noel Lucca, Galina Brychkova, Peter C. McKeown, Charles Spillane

**Affiliations:** Genetics and Biotechnology Lab, Plant and AgriBiosciences Research Centre (PABC), Ryan Institute, Aras de Brun, National University of Ireland Galway, University Road, Galway, H91 REW4 Ireland

**Keywords:** Plastid, Ribosome, Localization, Embryo development, *Arabidopsis thaliana*

## Abstract

Plastid ribosomal proteins (PRPs) can play essential roles in plastid ribosome functioning that affect plant function and development. However, the roles of many PRPs remain unknown, including elucidation of which PRPs are essential or display redundancy. Here, we report that the nuclear-encoded PLASTID RIBOSOMAL PROTEIN L5 (PRPL5) is essential for early embryo development in *A. thaliana*, as homozygous loss-of-function mutations in the *PRPL5* gene impairs chloroplast development and leads to embryo failure to develop past the globular stage. We confirmed the *prpl5* embryo-lethal phenotype by generating a mutant CRISPR/Cas9 line and by genetic complementation. As *PRPL5* underwent transfer to the nuclear genome early in the evolution of Embryophyta*,* PRPL5 can be expected to have acquired a chloroplast transit peptide. We identify and validate the presence of an N-terminal chloroplast transit peptide, but unexpectedly also confirm the presence of a conserved and functional Nuclear Localization Signal on the protein C-terminal end. This study highlights the fundamental role of the plastid translation machinery during the early stages of embryo development in plants and raises the possibility of additional roles of plastid ribosomal proteins in the nucleus.

**Supplementary Information:**

The online version contains supplementary material available at 10.1007/s00497-022-00440-9.

## Introduction

The plastid is an essential organelle in plant cells acquired through a unique endosymbiosis event in the common ancestor of all Archaeplastida, including green plants (Viridiplantae), in which a non-plastid eukaryote absorbed a photosynthetic bacterium (Kishino et al. 1990; Moreira et al. 2000; Stiller 2007; Nowack and Weber 2018). Gene transfer processes have occurred between the original plastid genome (of prokaryote origin) and the nuclear genome during the evolution of the photosynthetic eukaryotic cell leading most plastid-derived genes to relocate to the nucleus (Martin et al. 2002; McFadden 2014). This transfer is thought to have happened in parallel with the transfer of mitochondrial genes to the nucleus following the endosymbiosis event underlying eukaryotes. Since transferred genes include many of those responsible for the fundamental cellular and metabolic functions of the plastid their protein products needs to be targeted back to plastids by anterograde signaling to ensure their function (Bräutigam et al. 2007). Approximately two thirds of the Plastid Ribosomal Protein (PRPs) genes in the model eudicot *Arabidopsis thaliana* have been transferred from the plastid to the nucleus. Most of these genes have also been lost from the plastid genome with a small proportion still remaining and, thus, potentially redundant with their nuclear duplicates (Allen 2018).

Many prokaryote ribosomal proteins (RPs) have been shown to be essential in *E. coli* (Shoji et al. 2011), mostly being homologs of cyanobacteria-derived plastid RPs (Yamaguchi and Subramanian 2000). Similarly, many cyanobacteria-derived RPs have also been reported as essential for embryo development in at least one Embryophyta species (Table 1). Most essential PRPs are reported as necessary for embryogenesis in *A. thaliana* correlating with the essential functions of plastids in cellular metabolism before the start of photosynthetic activity, notably in lipid and starch biosynthesis (Neuhaus and Emes 2000). Genetic knockouts of these PRPs typically do not allow the embryo to develop further than the globular stage. This mutant phenotype highlights the necessity of the plastid translation mechanism as early as the globular stage of embryo development, and not before, even with the maternal-to-zygote transition occurring as early as the zygote (Zhao et al. 2019).

**Table 1:** Plastid ribosome subunits in *A.thaliana* and functional homologs in *E.coli*

PRP	Essentiality in *E. coli* (Shoji et al. 2011)	*A. thaliana* Locus ID	Essentiality post Globular Stage in *A. thaliana*	Reference (*A. thaliana*)
S1	Essential	AT5G30510	Non-essential	Romani et al. (2012)
S2	Essential	ATCG00160	Essential	Rogalski et al. (2008)
S3	Essential	ATCG00800	Essential	Fleischmann et al. (2011)
S4	Essential	ATCG00380	Essential	Rogalski et al. (2008)
S5	Essential	AT2G33800	Essential	Bryant et al. (2011); Muralla et al. (2011); Lloyd and Meinke (2012)
S7	Essential	ATCG00900/ ATCG01240	NA	NA
S8	Essential	ATCG00770	NA	NA
S9	Non-essential	AT1G74970	Essential	Hsu et al. (2010); Lloyd and Meinke (2012)
S10	Essential	AT3G13120	NA	NA
S11	Essential	ATCG00750	Essential	Muralla et al. (2011); Lloyd and Meinke (2012)
S12	Essential	ATCG00065/ ATCG01230	Putative essential	Asakura and Barkan (2006)
S13	Essential	AT5G14320	Essential	Bryant et al. (2011); Lloyd and Meinke (2012)
S14	Essential	ATCG00330	Essential	Jiang et al. (2018)
S15	NA	ATCG01120	Non-essential	Fleischmann et al. (2011)
S16	Essential	ATCG00050	Essential	Fleischmann et al. (2011)
S17	Non-essential	AT1G79850	Non-essential	Woo et al. 2002; Romani et al. (2012); Lloyd and Meinke (2012)
S18	Essential	ATCG00650	Essential	Rogalski et al. (2006)
S19	Essential	ATCG00820	NA	NA
S20	NA	AT3G15190	Essential	Romani et al. (2012)
S21	NA	AT3G27160	Non-essential	Morita-Yamamuro et al. (2004)
L1	NA	AT3G63490	Essential	Bryant et al. (2011); Romani et al. (2012); Lloyd and Meinke (2012)
L2	Essential	ATCG00830/ ATCG01310	NA	NA
L3	Essential	AT2G43030	NA	NA
L4	Essential	AT1G07320	Essential	Muralla et al. (2011); Lloyd and Meinke (2012)
L5	Essential	AT4G01310	Essential	This Study
L6	Essential	AT1G05190	Essential	Hsu et al. (2010); Muralla et al. (2011); Lloyd and Meinke (2012)
L7	Essential	NA	NA	NA
L9	NA	AT3G44890	NA	NA
L10	Essential	AT5G13510	Essential	Bryant et al. (2011); Lloyd and Meinke (2012)
L11	NA	AT1G32990	Non-essential	Pesaresi et al. 2006; Lloyd and Meinke (2012)
L12	Essential	AT3G27850	NA	NA
L13	Essential	AT1G78630	Essential	Hsu et al. (2010); Muralla et al. (2011); Lloyd and Meinke (2012)
L14	Essential	ATCG00780	NA	NA
L15	Non-essential	AT3G25920	Essential	Bobik et al. (2018)
L16	Essential	ATCG00790	NA	NA
L17	Essential	AT3G54210	NA	NA
L18	Essential	AT1G48350	Essential	Bryant et al. (2011); Lloyd and Meinke (2012)
L19	Essential	NA	NA	NA
L20	Essential	ATCG00660	Essential	Rogalski et al. (2008)
L21	Non-essential	AT1G35680	Essential	Yin et al. (2012)
L22	Essential	ATCG00810	Essential	Fleischmann et al. (2011)
L23	Essential	ATCG01300/ ATCG00840	Essential	Fleischmann et al. (2011)
L24	Non-essential	AT5G54600	Non-essential	Nadine et al. (2012); Romani et al. (2012)
L25	NA	NA	NA	NA
L27	Non-essential	AT5G40950	Essential	Romani et al. (2012)
L28	Essential	AT2G33450	Essential for greening process and post germination	Romani et al. (2012)
L29	Non-essential	AT5G65220	NA	NA
L30	Non-essential	NA	NA	NA
L31	NA	AT1G75350	Essential	Hsu et al. (2010); Lloyd and Meinke (2012)
L32	NA	ATCG01020	Essential	Fleischmann et al. (2011)
L33	NA	ATCG00640	Non-essential but affects growth in response to cold stress	Rogalski et al. (2008)
L34	Non-essential	AT1G29070	NA	NA
L35	NA	AT2G24090	Essential	Romani et al. (2012)
L36	NA	ATCG00760/ AT5G20180	Non-essential	Fleischmann et al. (2011)

Overview of experimentally demonstrated essentiality of Plastid Ribosomal Proteins (PRPs) for embryogenesis and cell survival in *A. thaliana.* The essentiality of plastid ribosomal proteins encoded in the plastid genome has also been demonstrated in *N. tabacum*. Each PRP is compared to its homologous ribosomal protein (RP) in the cyanobacteria-related species *E. coli*. The essentiality of RPs for cell survival in *E.coli * has been assessed by Shoji et al. (2011). *NA *not available (no data)

Some nuclear-encoded PRPs have been investigated in *A. thaliana* using genetic knockouts. PRPs in which loss-of-function mutations have been shown to lead to seed abortion have been considered as essential for embryo development and are summarized in Table 1. The essential requirement (essentiality) for plastid-encoded PRPs has been shown via knockout alleles in *Nicotiana tabacum* plastid genomes (biolistic chloroplast transformation) and considered essential based on leaf necrosis phenotypes. Among these *PRPS12* is putatively considered likely to be embryo lethal since the splicing of its mRNA is affected by the knockout of the gene *AtCAF2* (Asakura and Barkan 2006), but its essentiality for embryo development has not been directly demonstrated to date.

Notably, not all PRPs are reported to be essential for plant development including PRPS1, -S17, -L24 and -L28. Knockouts of *PRPS17* and *PRPL24* suggest they are non-essential. However *prps1-1* is shown to be only a knockdown so it could be considered that the viability of *prps1-1* seeds could be due to leaky expression in the mutant line which allows embryo development. Despite being non-essential for embryogenesis, *PRPL28,* however, appears to be required for seed greening at later stages of embryogenesis since its knockout creates albino seeds which are able to geminate but subsequently die quickly (Romani et al. 2012). A knockout of *PRPS17* has been shown to reduce growth rate as well as leaf chlorophyll pigment (Woo et al. 2002). *PRPL24* (Nadine et al. 2012), *PRPS21* and *PRPL11* knockouts also lead to decreased plant size and reduced photosynthetic activity due to a decrease in the translational activity in plastids (Morita-Yamamuro et al. 2004; Pesaresi et al. 2006). *PRPL33* was reported to be required only in cold-stress conditions (Rogalski et al. 2008), and *PRPS15* and *PRPL36* for full photosynthetic activity (Fleischmann et al. 2011).

Some homologs of the essential RPs in *E.coli,* however, still remain to be verified in green plants (PRPS2, -S4, -S7, -S8, -S10, -S11, -S12, -S19, -L2, -L3, -L16, -L17, -L19). However, non-essentiality in *E. coli* may not necessarily translate to non-essentiality in green plants. For example, while its homolog in *E.coli* was shown as non-essential, *PRPL15* was revealed to be essential for embryo development in *A. thaliana* (Bobik et al. 2018).

In this study, we demonstrate that PRPL5 is required for post-globular stage embryo development in *A. thaliana* and we characterize the activity its N-terminal Chloroplast transit peptide (cTP) and C-terminal Nuclear localisation signal (NLS) which are completely absent from Chlorophyta and Charophyta homologous proteins.

## Materials and methods

### Plant material and genomic samples

*Arabidopsis thaliana* seeds were surface sterilized with Chlorine gas (3:1 bleach:hydrochloric acid in a bell jar for one hour). Seeds were germinated on 0.5 × Murashige and Skoog (MS) medium (Murashige and Skoog 1962) containing 1% w/v sucrose and 0.8% w/v agar, and grown in a Percival Tissue Culture cabinet under a 16:8 h light: dark (21 °C/18 °C) regime (Boyes et al. 2001) until they were transferred to soil (five parts Westland compost [Dungannon, N. Ireland]: 1 part perlite: 1 part vermiculite). Plants were grown in chambers under fluorescent lamps at 200 μmol m^−2^ s^−1^ with the same photoperiod.

### Plant DNA extraction

Plant genomic DNA was extracted using 20 mg of rosette leaf which was grinded with glass beads and incubated in DNA extraction buffer [200 mM of Tris–HCl pH 7.5, 250 mM of NaCl, 25 mM of EDTA, 0.5% w/v of SDS] for 10 min at 60 °C. Samples were next mixed with equal volumes of ice cold isopropanol (1:1) and DNA was precipitated at  – 20 °C for 10 min followed by a centrifugation. Precipitated DNA was washed once with 70% ethanol, left over to dry, re-suspended in water and incubated at 60 °C for 10 min. To genotype plants, as wild type, heterozygous or homozygous, two different pairs of primers were used to span the insertion/removal site of the mutant alleles. The forward primer 5'-ATCCTCTCGAGGTAAGCGGT-3' and reverse primer 5'-TCTTCTCAGGTCGGTGTGGA-3' were used to span the T-DNA insertion from *prpl5-1* mutant allele, with the use of an additional internal reverse primer Lbb1.3 5'-ATTTTGCCGATTTCGGAAC-3' to detect the presence of the T-DNA sequence. The forward primer 5'-CACGCGCTAGCTTTTCACG-3' and reverse primer 5'-AGGGGCTAAACGGAAAACTCC-3' were used to span the Cas9-induced 1057 bp removal from *prpl5-2* mutant allele. In addition to the detection of a band of different size between wild-type and mutant alleles (1660 bp for WT and 603 bp for *prpl5-2*), one additional reverse primer 5'-TCCAAGGTGTGAGTCCCAGT-3' was used to detect the presence of the removed section from the wild-type allele.

## RNA extraction and PRPL5 expression analysis

RNA extraction was performed on 20 mg rosette leaf tissue using the ISOLATE II RNA Plant kit (Bioline, UK). DNase treatment was performed on 1 μg of crude RNA using the DNase I amplification grade kit (Invitrogen, UK), and cDNAs were generated using the Superscript III First Strand Synthesis kit (Invitrogen, UK), following protocol supplied by the manufacturer. *PRPL5* expression was evaluated by RT-PCR using forward 5'-TGGGTTTAATCAACAACGAACGC-3' and reverse 5'- TCCAAACCCTTGTCGTTCTG -3' primers and RT-qPCR using forward 5'-TCAACGCCTCAAAACCGCTT-3' and reverse 5'-TCCAAACCCTTGTCGTTCTG-3' primers. *PRPL5* Expression was normalized with *EF-1α* (Wang et al. 2014) using forward 5'-TCACCCTTGGTGTCAAGCAGAT-3' and reverse 5'-CAGGGTTGTATCCGACCTTCTT-3' primers.

### Thermal asymmetric interlaced (TAIL) PCR

Thermal asymmetric interlaced (TAIL) PCR was carried out according to (Liu et al. 2012). Three primers specific to the left border of pROK2 T-DNA were designed according to the publication specifications (the two first primers with overlapping sequences, and the third one at least 50 bp downstream): LB1 (5'-ATTTTGCCGATTTCGGAACCACC-3'), LB2 (5'-ACCATCAAACAGGATTTTCGCCTGCT -3'), and LB3 (5'-CCGTCTCACTGGTGAAAAGAAAAACC -3') and the same degenerative primers AD1 (5'-NTCGASTWTSGWGTT-3'), AD2 (5'-NGTCGASWGANAWGAA -3') and AD3 (5'-WGTGNAGWANCANAGA -3') have been used for all three reactions. Amplicons from the second reaction were extracted from a 2% w/v agarose gel, and sent for Sanger sequencing to LGC Genomics (Ireland). The resulting sequences were aligned with corresponding regions of the *Arabidopsis thaliana* genome (TAIR10.1) from the NCBI database.

### Cas9-directed mutagenesis of *Arabidopsis thaliana* Col-0

Cas9-directed mutagenesis was performed via the transformation of *A. thaliana* with a novel p3-Cas9-mcherry plasmid vector constructed in the SpillaneLab (Plasmid Map and Sequence in Supplementary Information). Briefly, the vector was constructed from the pORE03 backbone base (Coutu et al. 2007). First, a *BsaI* site from the Bar resistance gene was removed by converting A to G in the recognition site, conserving the encoded arginine residue; this was performed by *BsaI* and *HpaI* digestion followed by ligation of annealed oligos for sequence replacement. The backbone was then digested with *EcoRI* and *SphI* and ligated to the corresponding pHEE401 (Wang et al. 2015) fragment (a kind gift from Dr. Qi-Jun Chen’s lab, China Agricultural University, Beijing; Addgene plasmid #71,286; http://n2t.net/addgene:71286; RRID:Addgene_71286). A positive selectable marker was then inserted between the *NotI* and *SpeI* sites in the form of an *At2S3* (seed specific) promoter-driven mCherry gene block synthetized by Integrated DNA Technology (Leuven, Belgium), according to the protocol previously described (Gao et al. 2016).

**Fig. 1:**
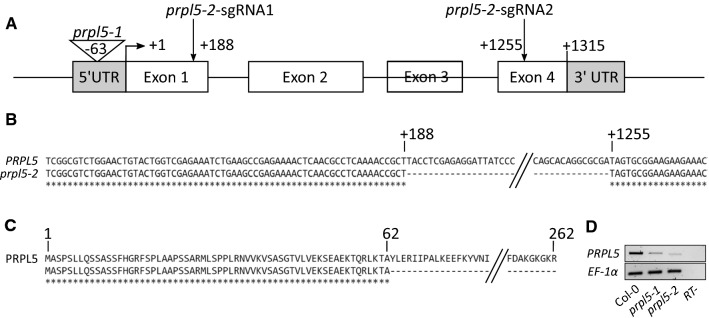
Mutant alleles of PRPL5 and expression data on mutant lines *prpl5-1* and *prpl5-2* used in this study. **A** Insertional mutant line *prpl5-1* and Cas9-generated mutant line *prpl5-2*. **B** Genomic sequence alignment between *PRPL5* WT sequence and *prpl5-2* mutant sequence validated by Sanger Sequencing. **C** Predicted amino-acid sequence of PRPL5 aligned with the predicted amino-acid sequence of PRPL5-2 mutant line. **D** RT-PCR of *PRPL5* expression in both *prpl5-1* and *prpl5-2*

The double guide system for Cas9-directed mutagenesis of At4g01310 was designed according to pre-established protocol (Xing et al. 2014) to remove a 1067 bp fragment from the gDNA sequence (Fig. 1a). Both guides were designed using the CRISPR-P tool (Lei et al. 2014) and the obtained double pair of primers were used to amplify the double guide promotor cassette from the vector pCBC-DT1T2 (gift from Qi-Jun Chen, Addgene plasmid #50,590), as described by Xing et al. (2014):

- DT1-BsF: 5’-ATATATGGTCTCGATTGCAGCACAGGCGCGATAGTGGTT-3’ and
- DT1-F0: 5’-TGCAGCACAGGCGCGATAGTGGTTTTAGAGCTAGAAATAGC-3’ (guide 1)
- DT2-BsR: 5’-ATTATTGGTCTCGAAACCTTACCTCGAGAGGATTATCAA-3’ and
- DT2-R0: 5’-AACCTTACCTCGAGAGGATTATCAATCTCTTAGTCGACTCTAC-3’ (guide 2)

The obtained amplicon was cloned into the p3-Cas9-mCheery vector using the Golden Gate reaction with *BsaI* restriction as described previously (Weber et al. 2011) with some modifications: one cycle of 5 min at 32 °C and 5 min at 16 °C for over 5 h, followed by 5 min at 50 °C and 10 min at 80 °C in a Applied Biosystems Veriti 96-well thermal cycler (Thermofisher Scientific, Paisley, UK).

The novel p3-Cas9-mcherry vector was used to transform *E.coli* DHα electrocompetent cells using electroporation. Cells were plated on LB agar with 50 µg/ml kanamycin, and incubated overnight at 37 °C. The vector was purified from a positive colony saturated culture solution using a plasmid extraction kit (Bioline, Dublin, Ireland), and the Cas9 cassette sequence was confirmed by Sanger sequencing (LGC genomics, Berlin, Germany). Electrocompetent cells of *Agrobacterium tumefaciens* strain GV3101 were transformed with the sequenced vector by the same method, and inoculated onto LB agar plates with 50 µg/ml kanamycin, 50 µg/ml rifampicin and 100 µg/ml gentamycin. Plates were incubated for two days at 28 °C.

Positive colonies were inoculated in LB broth with the same antibiotic concentrations for 2 days at 28 °C. Cells were then centrifuged and re-suspended in LB broth with 5% w/v sucrose and 0.02% v/v Silwett. This liquid culture was then used for *A. thaliana* flower dip method (Clough and Bent 1998), and seeds were harvested at the end of the plant life cycle.

Seeds were screened for transformants using the red fluorescence of the ERFP from the p3-Cas9-mcherry vector under green light, and positive T1 seedlings were grown on soil. Because no mutants were found within the T1 generation, seeds were harvested from T1 lines, screened for ERFP signal, and positive seeds were grown on soil. T2 seedlings were screened for Cas9-induced mutation by PCR, and positive bands were sent for Sanger sequencing and aligned against corresponding regions of the *A. thaliana* genome to confirm the removal of the sequence of interest.

### Cloning and transformation of *A. thaliana*

Genomic DNA sequences from *PRPL5* were amplified by Velocity DNA polymerase (Bioline, UK) using gateway-tailed primers and cloned first in pDONR221 and then in destination vector pB7YWG2 using the two-step gateway cloning system via BP and LR clonase reactions (Invitrogen, UK). Four different fragments were generated and cloned under the control of the 35S CamV promoter as fusion proteins with a C-terminal EYFP tag for fluorescence imaging: *PRPL5*, _Δ233-262_*PRPL5*, _Δ1-41_*PRPL5*, and _Δ(1–41)+(233–262)_*PRPL5*.

A set of four gateway-tailed primers were used: two primers for the full PRPL5 sequence (Forward 5'-attB1-ATGGCGTCTCCTTCGCTTC-3' and Reverse 5'-ATCTCTTTCCTTTTCCTTTAGCATCAAAG-attP1-3') and two for the N-ter and C-ter truncated sequence (Forward 5'-attB1-ATGGCGTCTGGAACTGTACTGGTC-3’ and Reverse 5'-ACCTGAAAGGCATTCCCATTAGAG-attP1-3'). One base was added to the reverse primers in 5' to keep *PRPL5* and *EYFP* in frame.

*A. thaliana* Col-0 lines were transformed by floral dip, as previously described. Resulting offspring were sprayed with 0.2 μg/ml of Basta to screen for transformants. Efficient transformation was verified by amplifying the YEFP sequence by PCR using forward 5'–3' and reverse 5'–3' primers and transformants with good fluorescence intensity were crossed with the chloroplast reporter line pt-ck (Nelson et al. 2007). Small pieces (1 cm square) of leaf tissue from offspring plants were mounted on a microscopic slide in 5 μg/ml DAPI in PBS buffer and visualized with an Olympus BX51 epifluorescence microscope (Dublin, Ireland) with an UV source X-cite Series 120 Q (EXFO, Knightwood, UK). Images were captured with a Leica DFC7000 T camera (Leica microsystems, Ashbourne, Ireland). The same vectors and *A. thaliana* transformation method were used to transform *prpl5-1* and *prpl5-2* mutants for the complementation rescue experiment.

### Sample preparation for transmission electron microscopy (TEM) analysis

Seeds were harvested at 4 days after pollination (DAP) from manually self-pollinated flowers from *prpl5-2* ± line and were fixed with a first solutions [2% v/v glutaraldehyde and 2% v/v paraformaldehyde in 0.1 M sodium cacodylate buffer pH 7.2] followed by a second solution [1% w/v osmium tetroxide in 0.1 M sodium cacodylate buffer pH 7.2] according to the procedure from NUI Galway Centre for Imaging. The seeds were then progressively dehydrated with increasing concentrations of ethanol up to 100%, washed in acetone, and finally embedded in resin using the Agar Low Viscosity Resin kit (Agar Scientific, Stansted, UK) according to the manufacturer’s protocol, and left for polymerisation at 65 °C for 48 h. Samples were cut using an ultramicrotome, survey sections of 500 nm width were cut using a glass knife, and stained with toluidine blue for microscope observation. From these, regions of interest were identified and trimmed to produce 70–90 nm sections using a diamond knife. Obtained sections were stained with uranyl acetate and lead citrate in the Leica EM AC20 automatic stainer (Leica microsystems, Ashbourne, Ireland) and allowed to air dry on a grid before visualization on a Hitachi 7500 Transmission electron microscope (Hitachi, Daresbury, UK).

### Identification of PRPL5 homolog sequences in Chlorophyta and Streptophyta

An initial search was performed by using the PRPL5 protein sequence from *A. thaliana* as query for a BLASTX search in Chlorophyta and Streptophyta (Mount 2007). The identified sequences were compiled and aligned using MUSCLE (version 3.5, Edgar (2004)) for phylogeny construction. The phylogeny was established using PhyML (version 3.0, Guindon et al. (2010)), and the phylogenetic tree was built using TreeDyn (version 196, Chevenet et al. (2006)).

## Results

### PRPL5 is essential for seed development in *Arabidopsis thaliana*

**Fig. 2:**
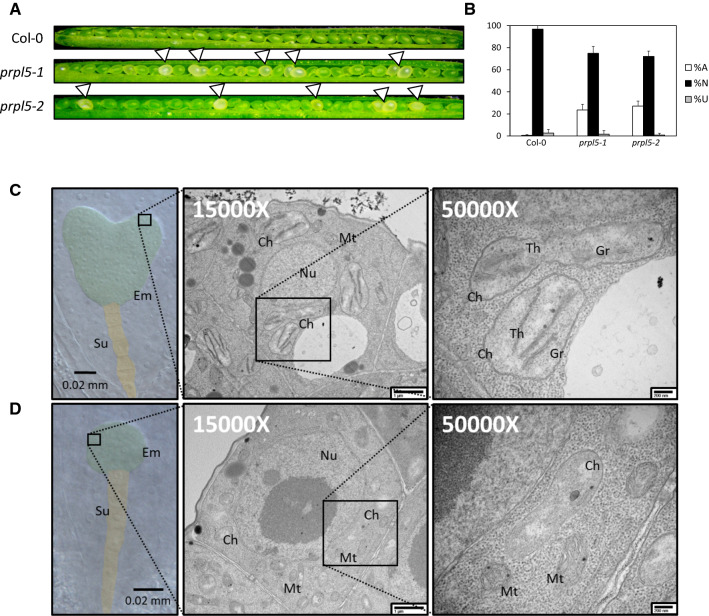
Heterozygous mutant of *AT4G01310* leads to 25% seed abortion phenotype. Seed and plastid phenotype observed in both mutant lines in comparison to WT Col-0. **A** Developing seeds in siliques at 7 DAP. Aborting seeds are white and indicated by arrows. **B** Percentage of ANU (Aborted, Normal and Unfertilized) seeds for WT, *prp5-1* and *prpl5-2* lines. **C** Green seed embryo from *prpl5-*2 at 4 DAP (heart stage) seen in false colors. The embryo has been highlighted in green while the suspensor has been highlighted in yellow. Chloroplasts as fully developed as shown on TEM pictures aside, with presence of thylakoid and grana in the chloroplast matrix. **D** White seed embryo from *prpl5-2* at 4 DAP (arrested at globular stage) seen in the same false colors as above. Chloroplasts are under-developed as shown on TEM pictures aside, with no thylakoid nor grana being observed in the plastid matrix. *Em* Embryo, *Su* Suspensor, *Ch* Chloroplast, *Nu* Nucleus, *Mt* Mitochondria, *Th* Thylakoid, *Gr* Granum

In a forward genetic screen for seed abortion mutants, we observed a 23.5% seed abortion mutant phenotype in the T-DNA line SALK_015079 which was not segregating with the mutations on *AT3G59380* reported in the line by the Nottingham Arabidopsis Stock Centre (Fig. 2A and B). To identify the gene associated with the seed abortion phenotype, we used a TAIL-PCR approach on DNA extracted from a plant harboring the phenotype, which revealed the presence of a so far unreported pROK2 T-DNA insertion in the 5’UTR region of *AT4G01310* (Fig. 1A), which codes for the PLASTID RIBOSOMAL PROTEIN L5 (PRPL5) according to previous proteomic characterization (Zybailov et al. 2008; Ferro et al. 2010).

The genetic segregation of the insertion in *AT4G01310* (*PRPL5*) in the F1 offspring of a self-fertilized heterozygous parent followed a 1:2 ratio between genotyped wild-type plants and heterozygous mutants (χ^2^ test on 20 plants, *p*-value = 0.7903), with the total absence of any homozygous mutant offspring. The presence of the mutation in *PRPL5* correlated exclusively with the presence of the seed abortion mutant phenotype. To confirm the causality of the insertion on the phenotype, we performed a Cas9-directed mutagenesis on wild-type (WT) Col-0 line using the double guide targeting method (Xing et al. 2014) with a p3-Cas9-mcherry vector to generate a 1057 bp deletion in the *PRPL5* genomic sequence (Fig. 1A and B). Transformants were isolated from T2 generation seed by Enhanced Red Fluorescent protein (ERFP) fluorescence and the presence of a mutant allele of *PRPL5* was verified by Sanger sequencing across the deleted region. This verification further demonstrated that the 1057 bp removal created a stop codon immediately after the 5’ Cas9 cut (Fig. 1C). The offspring were then harvested and transgene-free lines were isolated by screening for non-fluorescent seeds. A 27.0% seed abortion phenotype was observed from this purified line, agreeing with the segregation ratio previously observed (Fig. 2A and B). Again, with this Cas9-generated *prpl5* mutant line, no homozygous mutant could be isolated from the offspring and the presence of the deletion mutation in *PRPL5* followed a 1:2 distribution for WT and heterozygous mutants (χ^2^ test on 60 plants, *p*-value = 0.7589) and correlated with the presence of the seed abortion phenotype. Since no mutants of *PRPL5* have so far been reported in the scientific literature, the two mutant alleles described in this study are hereafter named *prpl5-1* (SALK line) and *prpl5-2* (Cas9-mutated line). *PRPL5* mRNA expression was quantified by semi-quantitative RT-PCR (Fig. 1D) using a pair of primers spanning the locus for both *prpl5-1* and *prpl5-2* mutations, where any mutant allele mRNA from either *prpl5-1* or *prpl5-2* mutant lines would not amplify. As both of these mutant lines are heterozygous, we expected a decrease of *PRPL5* expression in both the *prpl5-1* and *prpl5-2* lines in comparison to PRPL5 expression levels in wild-type Col-0, since one of the two *PRPL5* alleles in each heterozygous line was genotyped as wild type. The semi-quantitative RT-PCR results confirm that the heterozygous lines of *prpl5-1* and *prpl5-2* display reduced expression levels as expected from lines with only one wild-type copy.

### Lack of PRPL5 function causes post-globular embryo arrest

Seeds from siliques collected from self-pollinated *prpl5-2* heterozygous mutant plants were extracted at different timepoints from 1 to 6 days after Pollination (DAP) and cleared. Compared to wild type, normal embryogenesis was observed in all developing seeds from the heterozygous mutant parent up to 3 DAP. After this timepoint, the embryos of 72.1% of the progeny seeds continue to develop normally (Fig. 2C). However, the other 27% of developing seeds remained white after this timepoint (identified as homozygous mutants) and displayed a post-globular stage embryo arrest phenotype (Fig. 2D). The remaining 0.9% of the ovules were unfertilized.

To determine whether the embryo abortion phenotype is associated with aberrant plastid development, progeny seeds from siliques obtained from self-pollinated *prpl5-2* heterozygous mutants were extracted at 4 DAP and processed for Transmission Electron Microscopy (TEM). Phenotypically normal plastids were observed in the normally developing embryos (green seeds), including the presence of fully developed thylakoids and grana (Fig. 2C). In contrast, while the plastid double envelope was still visible in post-globular arrested (homozygous mutant) embryos, the plastids were less than half the size of wild-type plastids and completely lacked any thylakoids or grana (Fig. 2D). Hence, we demonstrate that a homozygous mutation of *PRPL5* leads to defective plastid development causing embryo arrest at the globular stage and subsequent seed abortion.

### The PRPL5 N-terminal peptide sequence is necessary for plastid localization, while the C-terminal peptide sequence is not

**Fig. 3:**
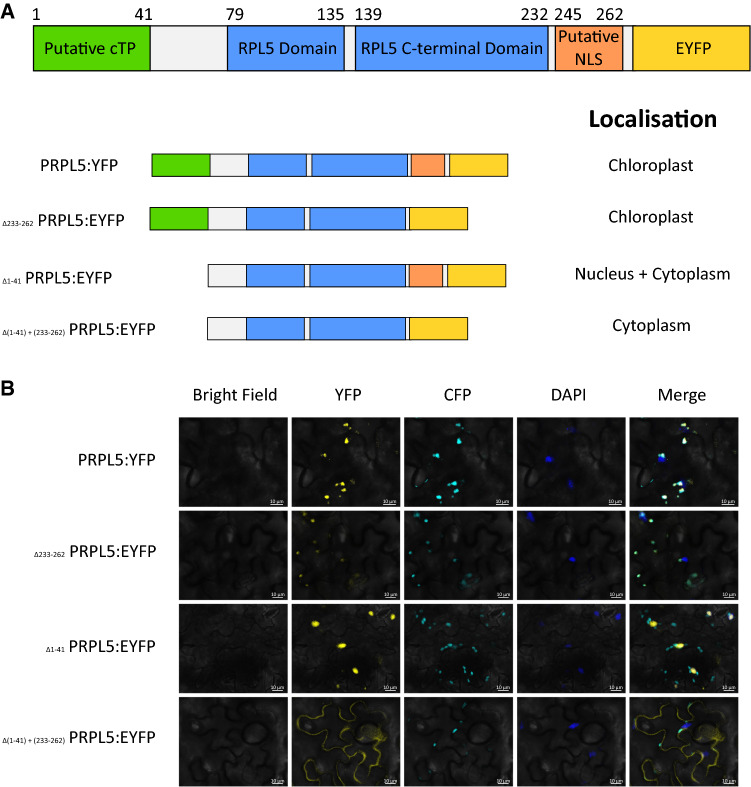
Subcellular localization of PRPL5:YFP protein constructs in *A. thaliana* leaf epidermal cells. Subcellular localization of four different PRPL5:YFP construct within *A. thaliana* leaf abaxial cells to interrogate the localization functionality of predicted N-ter and C-ter signaling peptides. **A** C-terminal fusion protein of PRPL5 with EYFP (Venus). Description of the localisation of each fusion protein: PRPL5:EYFP (Full protein positive control), _Δ233-262_ PRPL5:EYFP (C-terminal truncated protein), _Δ1-41_ PRPL5:EYFP (N-terminal truncated protein, and _Δ(1–41)+(233–262)_ PRPL5:EYFP (N-ter and C-ter truncated negative control). **B** EYFP visualization of protein localization. PRPL5:EYFP and _Δ233-262_ PRPL5:EYFP co-localize with chloroplast reporting signal, _Δ1-41_ PRPL5:EYFP co-localize with DAPI (nucleus), and _Δ(1–41)+(233–262)_ PRPL5:EYFP localizes only in the cytoplasm. Nucleus localisation is displayed using DAPI and chloroplasts localisation is displayed using a CFP construct inherited from the *A. thaliana* pt-ck reporter line. White bar represents 10 μm

As the nuclear-encoded *PRPL5* gene is essential for plastid development, its protein product must be targeted to the plastid by anterograde signaling early during embryogenesis. Hence, we sought to identify the molecular basis of its intracellular targeting. Bioinformatic analysis of the PRPL5 peptide sequence with ChloroP (Emanuelsson et al. 1999) predicted amino acids 1 to 39 to form a chloroplast transit peptide (cTP) with relative certainty (Score 0.575). In contrast, PROSITE (Sigrist et al. 2002) and cNLS mapper predictor (Kosugi et al. 2009) identified amino acids 245–262 to form a bipartite nuclear localization signal with a middle level score of 3.7 for cNLS mapper (Fig. 3A). Interestingly, analysis of the Cryo-EM structure of the *Spinacea oleracea* plastid ribosome (Perez Boerema et al. 2018) indicates that the amino acids 1–41 at the N-terminus and from 233 onwards at the C-terminus sequences are absent from the protein once incorporated into the plastid ribosome (Fig. 3A). This suggests that the nascent PRPL5 polypeptide contains multiple intracellular targeting sequences. To investigate these targeting sequences and determine their intracellular localisation functionality, we used the cleavage sites identified in PRPL5 from *S. oleracea*, which are very close to the one in *A. thaliana*, to delimit both N-terminal and C-terminal sequences and to investigate their effect on PRPL5 intracellular localisation.

To interrogate the intracellular localisation functionality of different PRPL5 peptide regions, four constructs were generated from the *PRPL5* wild-type sequence and used to stably transform Arabidopsis Col-0 plants. The first construct consisted of a C-terminal fusion of PRPL5 to EYFP (i.e., p35S:*PRPL5*:*EYFP*) and was used as a positive control for PRPL5 protein localisation. The other three constructs were designed to lack either the N-terminal peptide sequence (i.e., p35S:_Δ1-41_*PRPL5*:*EYFP*), the C-terminal peptide sequence (i.e., p35S:_Δ233-262_*PRPL5*:*EYFP*), or both (i.e., p35S:_Δ(1–41)+(233–262)_*PRPL5*:*EYFP*). These constructs were used to stably transform *A. thaliana* Col-0 and transformants with good fluorescence intensity were crossed with the chloroplast reporter line pt-ck (Nelson et al. 2007). To visualize intracellular localization, leaf tissue was mounted on microscopic slide with a 5 μg.L^−1^ DAPI solution for fluorescence imaging.

The positive control PRPL5:EYFP protein construct co-localized with chloroplasts, as expected for a nuclear-encoded plastid protein (Fig. 3A). Such co-localization with chloroplast also was observed with _Δ233-262_PRPL5:EYFP lacking the C-terminal targeting sequence (Fig. 3B). However, the protein _Δ1-41_PRPL5:EYFP, which lacks the N-terminal sequence, did not co-localize with chloroplasts but rather localized within the cytosol, as well as in the nucleus (Fig. 3C). Finally, the _Δ(1–41)+(233–262)_PRPL5:EYFP construct lacking both N- and C-terminal peptide sequences localized exclusively in the cytosol (Fig. 3D). Overall, these results indicate that both predicted targeting sequences are functional although localization of PRPL5 to the nucleus only occurs in the absence of the N-terminal cTP sequence.

### The cTP of PRPL5 is essential for complementation of the *prpl5-1* and *prpl5-2* mutant phenotypes

**Fig. 4:**
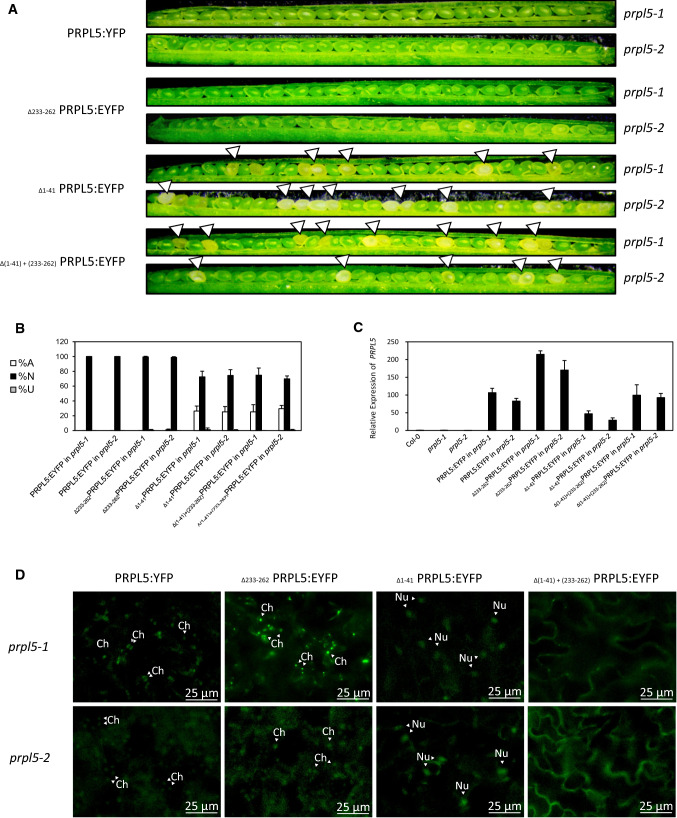
Genetic complementation rescue experiment of *prpl5-1* and *prpl5-2*. Seed phenotype of mutant lines *prpl5-1* and *prpl5-2* rescued with different constructs of PRPL5:YFP harboring no, one, or both N-ter and C-ter signaling peptides. **A** Developing seeds in 7 DAP siliques from transformed *prpl5-1* and *prpl5-2* lines. **B** % of unfertilized ovules (U), and normal and aborted seeds (N, A), for transformed *prp5-1* and *prpl5-2* lines. **C** RT-qPCR of p35S:*PRPL5:YFP* in leaves of transformed *prpl5-1* and *prpl5-2* plants. **D** PRPL5:YFP fluorescence in rescued mutant lines. *Ch* Chloroplast, *Nu* Nucleus

The PRPL5 fusion and truncated protein constructs were used in complementation assays for the 25% seed abortion phenotype (observable in *prpl5-1* and *prpl5-2* heterozygotes) to confirm that *PRPL5* is responsible for the observed abortion phenotype and also to interrogate the functions of the targeting peptides in relation to the seed abortion phenotype (Fig. 4). Both *prpl5-1* and *prpl5-2* heterozygous mutants were stably transformed with each of the four constructs by floral dipping and transformants were selected with Basta. The percentages of fertilized and unfertilized ovules and aborted and normal seeds were assessed for each transformant as previously reported to assess both fertility and viability (Duszynska et al. 2019). The expression of each transgene was also verified by both YFP fluorescence and RT-qPCR. Both the PRPL5:EYFP and _Δ233-262_PRPL5:EYFP constructs were able to fully restore the WT phenotype in both of the heterozygous mutant lines, whereas the heterozygous mutants transformed with _Δ1-41_PRPL5:EYFP and _Δ(1–41)+(233–262)_PRPL5:EYFP constructs still displayed 25% seed abortion (Fig. 4B) despite strong expression of the transgenes (Fig. 4C and D). Therefore, the constructs with the N-terminal deletion are unable to complement the *prpl5* loss-of-function alleles, as expected given their lack of plastid localisation. The genotyping of the T2 complemented generation further revealed the presence of homozygous mutants for both *prpl5-1* and *prpl5-2* complemented by PRPL5:EYFP and _Δ233-262_PRPL5:EYFP, demonstrating the capability of both constructs to rescue PPRL5 function. Therefore, plastid localisation of PRPL5 with a cTP sequence is required for *A. thaliana* embryogenesis, while nuclear localisation is not required despite the NLS being functional.

### PRPL5 transitioned from plastid-encoded to nuclear-encoded in the common ancestor of embryophytes

**Fig. 5:**
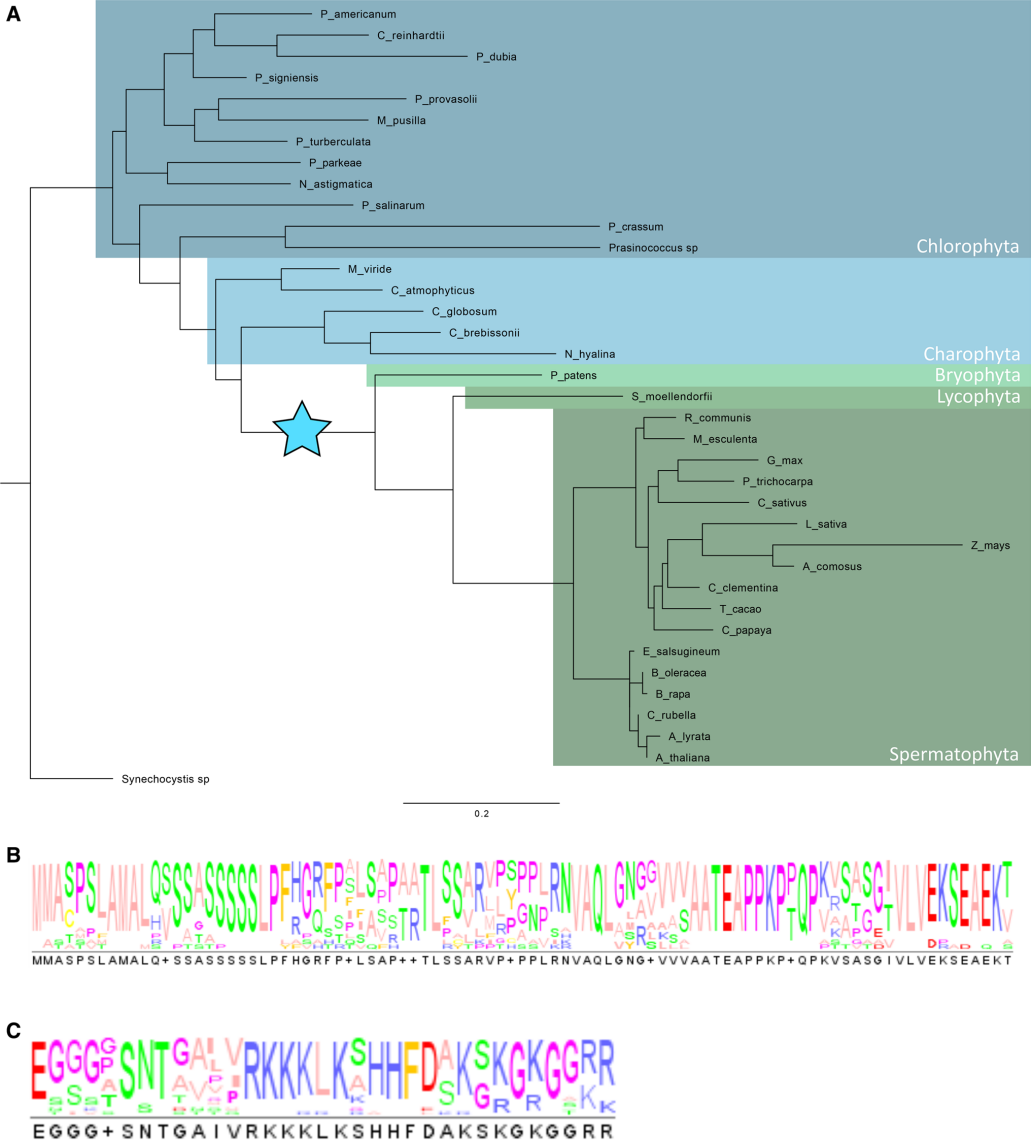
Evolution of PRPL5 across *Chlorophyta* and *Embryophyta*. Study of PRPL5 molecular evolution in Viridiplantae and conservation of N-ter and C-ter signaling peptides across species **A** Phylogenetic tree of the PRPL5 sequence in Viridiplantae. The tree is rooted on the cyanobacteria species *Synechocystis sp.* (RPLE). Blue star indicates the interval in which *PRPL5* gene underwent transfer to the nuclear genome and corresponding loss from the plastid genome. **B** Consensus sequence of PRPL5 N-terminal signaling peptides among species displayed in the tree. **C** Consensus sequence of PRPL5 C-terminal signaling peptides among species displayed in the tree

To gain evolutionary insights into the function of both N-terminal and C-terminal signaling peptides of PRPL5 we investigated the origin of PRPL5 through the evolution of Viridiplantae. From the comparison between PRPL5 sequences from Chlorophytes to spermatophytes, we found that the PRPL5 protein is exclusively nuclear-encoded in every sequenced species of the Embryophyta clade (Fig. 5). Both the N-terminal and C-terminal sequences of PRPL5 are well conserved in all seed plants (Spermatophyta) with circa 40% sequence identity for the N-terminal sequence, and circa 60% sequence identity for the C-terminal sequence. When compared to *A. thaliana,* the PRPL5 sequences of *Zea mays* and *Ananas comosus* were found to be the most distant with only 28% sequence identity to *A. thaliana* for the N-terminal sequence and 50% for the C-terminal, suggesting some divergence of PRPL5 sequence variation in monocots. The first and last 10 amino acids of the N-terminal sequence are the most conserved, while an RKK_LK_HHF__K_KG motif in the C-terminal sequence is also extremely well conserved across all analyzed sequences from Embryophyta.

All other groups from Chlorophyta and Charophyta (all Streptophyta species except land plants (Petersen et al. 2006)) have *PRPL5* exclusively encoded in their plastid genomes, indicating that the gene underwent transfer to the nuclear genome in the common ancestor of Embryophytes (indicated with a blue star in Fig. 5). No conserved N-terminal nor C-terminal sequences comparable to those in Embryophyta can be detected in the PRPL5 sequence from any Chlorophyta or Charophyta species. The protein sequences of PRPL5 found in Lycophyta and Bryophyta both possess an N-terminal and C-terminal signaling peptide, but these are not well conserved comparative to the ones found in Spermatophyta species. We find only 22% identity for the N-terminal sequence from Physcomitrium patens when compared the one from A. thaliana, and 18% identity for Selaginella moellendorfii. The C-terminal sequence also harbors only 20% and 24% identity for *P. patens* and *S. moellendorfii*, respectively, in comparison to *A. thaliana*. In addition, both the N-terminal and C-terminal sequence tracts appear to be longer in both species in comparison to A. thaliana (53 and 54 amino acids for P. patens and S. moellendorfii, respectively, compared to 39 amino acids for Spermatophyta species), with the C-terminal section also lacking the Spermatophyta conserved motif. Despite these differences, ChloroP predicts N-terminal sequences as cTPs with a similar certainty as for the *A. thaliana* PRPL5 sequence (scoring at 0.505 for *S. moellendorfii*, 0.581 for *P. patens*). The C-terminal sequence is also predicted by cNLS mapper predictor as being a bipartite NLS in *S. moellendorfii* and *P. patens*, albeit with less certainty than for *A. thaliana* (scores of 2.0 for both). Thus, the transition of PRPL5 from being plastid-encoded to nuclear-encoded after the divergence of Embryophytes from Charophytes can be associated with the co-apparition of both plastid and nuclear localization signals.

## Discussion

### PRPL5 is required for plastid development

The bacterial homolog of *A. thaliana* PRPL5 (i.e., RPLE) has been reported as essential in *E.coli* (Shoji et al. 2011), but any requirement of PRPL5 for plant function has not previously been determined. As previously observed with most essential PRPs, a lack of functional PRPL5 does not allow any embryo development past the globular stage and causes embryo cells to proliferate ectopically while cotyledon initiation fails to occur. We demonstrate by TEM imaging that no thylakoids develop in plastids from either *prpl5-1* or *prpl5-2* homozygous mutants, consistent with the smaller and under-developed plastid phenotype reported in knockouts mutants of *PRPL21* (Yin et al. 2012). This plastid requirement for normal embryogenesis is also observed across different protein pathways. The knockout of the plastid Glycyl t-RNA transferase *EDD1* also leads to embryo failure, highlighting the importance of protein translation in plastids at such an early stage (Uwer et al. 1998). The same embryo phenotype can be observed with the knockout of *DLC* (Bellaoui et al. 2003) and the Stromal Processing Peptidase gene *SPP* (Trösch and Jarvis 2011) intervening in the cleavage of plastid transit peptides. More recently, two partially redundant nuclear-encoded chloroplast proteins for growth and fertility (genes *CGF1* and *CGF2*) have been reported as embryo-defective, as well as being required for normal female gametogenesis (Zhu et al. 2020). This underlines the importance of anterograde protein signaling for the functioning of chloroplasts and the role of chloroplast during early embryogenesis.

An explanation for the requirement of a well-functioning plastid at such an early stage of embryo development (prior to any photosynthetic activity) can be derived from the essentiality of plastid-derived lipid and starch biosynthesis pathways (Neuhaus and Emes 2000). One the genes involved in such pathways is *accD* (which encodes a plastid acetyl-CoA Carboxylase and is essential for embryogenesis (Kode et al. 2005)), remains located within the plastid genome in *A. thaliana* while all the other genes related to the same pathways have already been transferred to the nuclear genome during evolution. The plastid genome haboring the only copy of *accD* in *A. thaliana* contrasts with other species (such as *Z. mays* (Bryant et al. 2011) and different species from the *Campanulaceae* linage (Rousseau-Gueutin et al. 2013)), *accD* homologues have undergone transfer to the nuclear genome. In comparison to *A. thaliana*, impairment of the plastid translation machinery does not lead to embryo defect phenotypes in *Z. mays* but only to an impairment of the greening process (Asakura and Barkan 2006; Bryant et al. 2011). This suggest that control of the lipid and starch biosynthesis pathways has been completely transferred over to the nucleus in these species and does not need plastid-related translation anymore to function, while in *A. thaliana* a part of this control is still shared between nucleus and plastid which jeopardizes its functionality in the event of plastid malfunction.

### PRPL5 may play a core role in the cohesion of the plastid ribosome

It must be noted that, even though most PRPs are required for embryo development, there are also many PRP exceptions to this expectation. It is likely that some PRPs are more important than others for plastid ribosome activity depending on their position and functional role within the ribosome structure. As such, the necessity of PRPL5 can be assessed from investigation of the different interactions it can have within the whole quaternary protein complex. First, PRPL5 is known to form a heterodimer with PRPL11, this heterodimer binding to the 5S RNA to form one of the core elements of the large ribosome subunit (Steitz et al. 1988; Pelava et al. 2016). Second, the recent Cryo-EM structure of the spinach chloroplast ribosome shows that PRPL5 binds to RPL31, forming what is termed the central protuberance of the 50S ribosomal subunit. PRPL5 further is involved in a bridge between small and large ribosomal subunits via an interaction with PRPS13, an interaction strengthened by the association of the Ribosomal pY factor (Ahmed et al. 2016; Bieri et al. 2017; Perez Boerema et al. 2018). Despite not being the only bridge structure observed between both ribosomal subunits, PRPS13, however, appears to be required for plastid ribosome normal function, as mutations of *PRPS13* have also been reported as embryo-defective (Bryant et al. 2011; Lloyd and Meinke 2012). The maintenance of the large subunit central protuberance also seems to be required for ribosome normal activity, as *PRPL31* was also reported as essential for embryogenesis (Hsu et al. 2010). Overall, this suggests a central position for PRPL5 in the structural organization of the plastid ribosomal complex.

### Roles of N-terminal and C-terminal peptide sequences in PRPL5

In this study we functionally demonstrate that the first 41 amino acids of PRPL5 act as a cTP (matching the predictions made by ChloroP). The plastid localization of the PRPL5 protein is also supported by mass spectrometry data from isolated chloroplasts (Kleffmann et al. 2004) and further reports added to the PPDB (Zybailov et al. 2008) and AT_CHLORO proteomic databases (Ferro et al. 2010). The N-terminal sequence of PRPL5 is well conserved among land plants and seems to be exclusively responsible for PRPL5 localization within chloroplasts. The presence of the C-terminal peptide in PRPL5 leads to a localization of the protein within the nucleus, confirming PROSITE and cNLS mapper predictions. However, we report that such nuclear localization of PRPL5 only occurs when the N-terminal cTP is disrupted, suggesting that the cTP function is dominant over the NLS one. Since truncated version of PRPL5 lacking the C-terminal NLS sequence can still complement both mutant lines used in this study, it is unclear what role(s) the NLS peptide may play in PRPL5 function.

Comparisons with other nuclear-encoded PRPs also suggests that a C-terminal NLS can be predicted in the majority of PRPs. Only in PRPS1, -L4, -L17 and -L18 can no NLS be predicted. A recent study has demonstrated that the acquisition of a cTP on nascent plastid targeted proteins can usually be attributed to insertions or deletions in nearby genome sequences, followed by substitutions at a lower rate (Christian et al. 2020). Acquisition of a cTP by gene duplication is considered a less likely scenario, which could explain the weak alignment of cTP sequences from different nuclear-encoded PRPs within *A. thaliana* (Supplementary Fig. S5A). The same observation can be made with respect to the C-terminal NLS predicted in the majority of PRPs (Supplementary Fig. S5B). Hence, we can likely infer that cTPs and NLSs of the different nuclear-encoded PRPs were obtained from different origins.

However, in comparison to the importance of a cTP for the function of such proteins, which would explain its strong conservation across plant lineages (Fig. 5B), the high level of conservation of an NLS is puzzling (Fig. 5C). Such a high level of conservation would indicate a conservation of its function across plant lineages, but we could not identify any obvious phenotype in mutant lines rescued with a truncated version of PRPL5 lacking the C-terminal NLS under normal growth conditions. An interesting mechanism which could explain the role of PRPL5 C-terminal NLS would be the relocation of protein in the nucleus to perform a different function under stress conditions, possibly to be recruited by RNA polymerases to maintain genome integrity. This would be possible without the intervention of alternative splicing or post translational modifications, using a mechanism which has been named moonlighting and which uses close contacts between organelles and nucleus to allow such a transfer (Foyer et al. 2020; Krupinska et al. 2020). So far, PRPL5 has been identified in only one crude nuclear lamina protein fraction isolated from *A. thaliana* leaf-derived protoplast (Sakamoto and Takagi 2013) which could correlate with a nuclear relocation under particular stress conditions. Protoplasts indeed harbor an atypical and somewhat artificial cellular environment as cells are disconnected from their usual tissue. The process of protoplast isolation has also been reported to induce an oxidative burst and the activation of oxidative stress responses genes, with an intensity varying between plant species (Papadakis and Roubelakis-Angelakis 1999).

Other PRPs have also been detected in nuclear environments in published nuclear proteomes. For instance, Bae et al. (2003) report the detection of PRPS5 and of a protein identified as a homolog of PRPL21 within a whole leaf tissue nuclear proteins extract. Both proteins were, however, identified as less present in the nuclear environment after application of cold stress. Similarly, Goto et al. (2019) report the presence of PRPS5, -S17 and -L21 in the proteome extracted from cultured cells nuclei, while Sakamoto and Takagi (2013) reports the presence of 24 PRPs in the proteome of protoplast nuclei in addition to PRPL5, namely PRPS1, -S5, -S9, -S13, -S17, -S20, -L1, -L3, -L4, -L6, -L9, -L10, -L11, -L13, -L15, -L17, -L18, -L21, -L24, -L27, -L28, -L29, -L31, and -L35. Such findings may link plastid protein relocation to the nucleus in the particular case of intense stress conditions and might explain the reported impact of some PRPs mutations on plant development only in stressful conditions, such as for PRPL33 (Rogalski et al. 2008). Hence, we could consider that PRPL5 could relocate in the nucleus under such conditions. However, any function of PRPL5 in the nuclear compartment is yet to be determined.

## Conclusions

We demonstrate in this study that a protein of the central protuberance of the plastid 50S ribosomal subunit, namely PRPL5, is required for embryogenesis past the globular stage in *A. thaliana* indicating that PRPL5 is critical for the function of the plastid ribosome, and hence for translation. We further demonstrate that the N-terminal and C-terminal ends of the PRPL5 protein function, respectively, as a cTP and NLS, and further elucidate that the cTP is critical for protein function, while the NLS of PRPL5 is not. Despite its functionality as an NLS, the functional role and the conditions upon which this NLS signal is relevant to plant development and growth remains to be determined. The fact that this NLS signal is well conserved in all of its land plant homologues (especially in the seed plants) suggests some functional significance. Overall, our study identifies an essential role for the plastid 50S ribosomal subunit PRPL5 in embryogenesis and defines the functional cTP of the protein, while raising questions regarding the functional role of the predicted NLSs present in most nuclear-encoded plastid ribosomal proteins.

## Supplementary Information

Below is the link to the electronic supplementary material.

Supplementary file1 (DOCX 727 kb)

## Data Availability

None.
