# Supplementary material for: Plastid ribosome protein L5 is essential for post-globular embryo development in Arabidopsis thaliana
Source: Plant Reprod. 2022 Mar 5;35(3):189–204. doi: 10.1007/s00497-022-00440-9 (PMC9352626; doi:10.1007/s00497-022-00440-9)
Supplement: Supplementary file 1 — Supplementary file1 (DOCX 727 kb) [file 497_2022_440_MOESM1_ESM.docx]

**Supplementary Information**


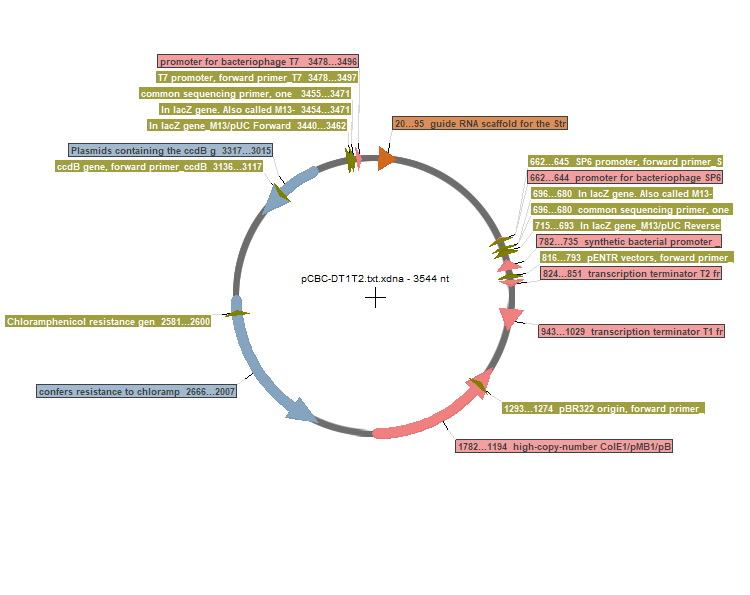


**Supplementary Fig. S5.1** Map of the Cas9 cassette plasmid pCBC-DT1T2


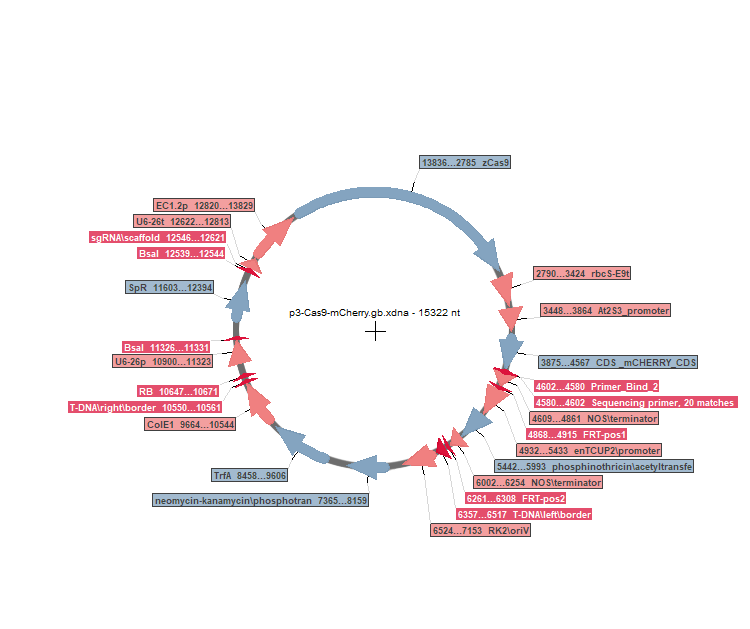


**Supplementary Fig. S5.2** Map of the novel p3-mcherry plasmid vector used for Cas9-directed mutagenesis on Col-0 to generate the *prpl5-2* mutant line.


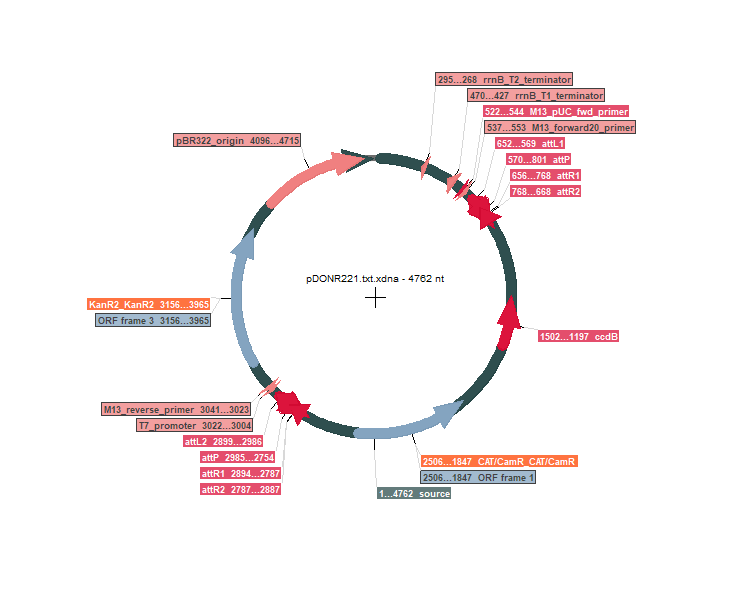


**Supplementary Fig. S5.3** Map of Gateway pDONR221 vector.


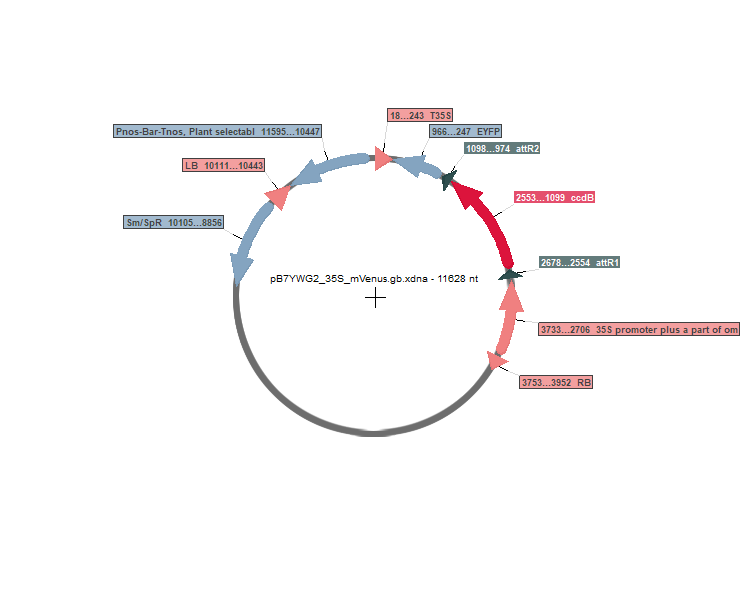


**Supplementary Fig. S5.4** Map of pBYYWG2 used for as destination vector for subcellular localisation of PRPL5:EYFP.


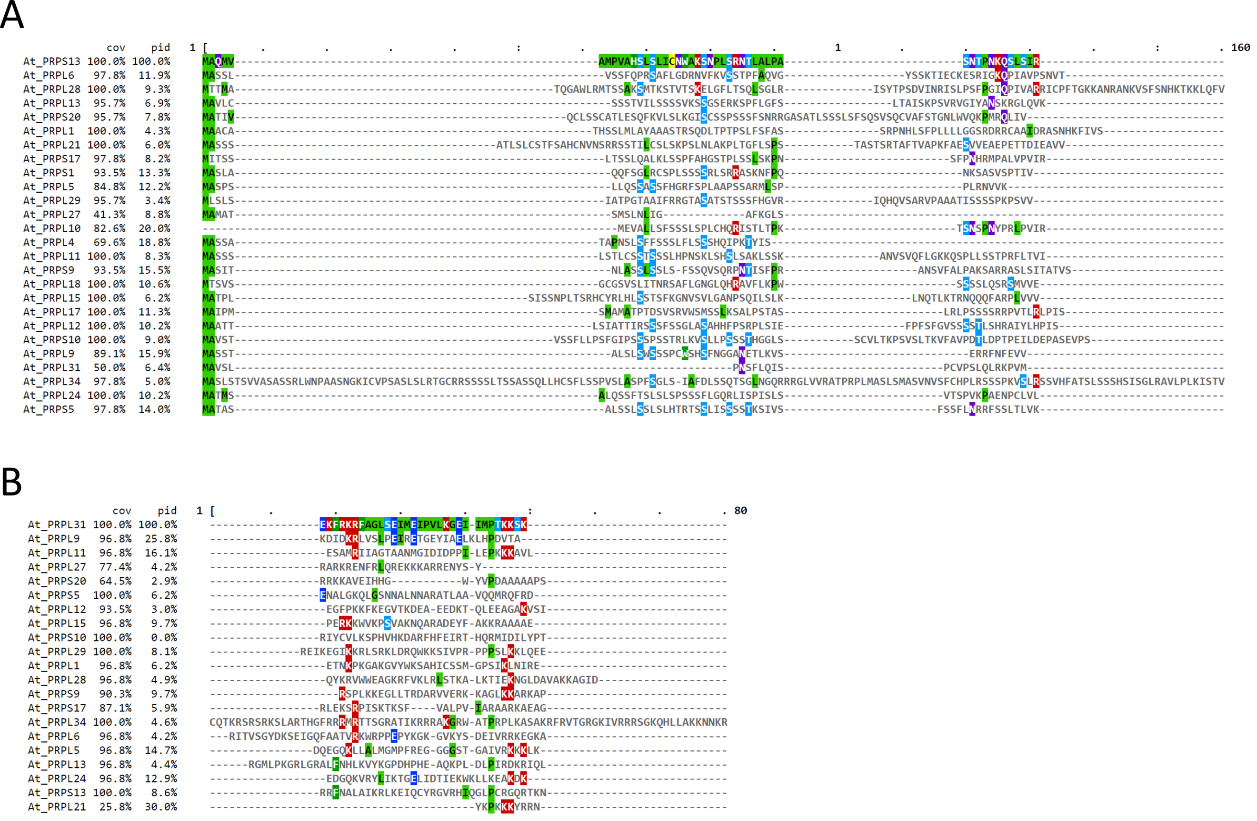


**Supplementary Fig. S5.5** Alignment of predicted cTP (A) and NLS (B) sequences in nuclear encoded PRPs.
